# Human dental pulp stem cells can differentiate into Schwann cells and promote and guide neurite outgrowth in an aligned tissue-engineered collagen construct *in vitro*

**DOI:** 10.1096/fj.13-243980

**Published:** 2014-04

**Authors:** Wendy Martens, Kathleen Sanen, Melanie Georgiou, Tom Struys, Annelies Bronckaers, Marcel Ameloot, James Phillips, Ivo Lambrichts

**Affiliations:** * Department of Functional Morphology and; † Department of Biophysics, Biomedical Research Institute (BIOMED), Hasselt University, Diepenbeek, Belgium;; ‡ Department of Life, Health, and Chemical Sciences, The Open University, Milton Keynes, UK;; § Department of Biochemical Engineering and; ‖ Department of Biomaterials and Tissue Engineering, Eastman Dental Institute, University College London, London, UK

**Keywords:** neural regeneration, nerve repair, glial cell, myelination, cellular hydrogel

## Abstract

In the present study, we evaluated the differentiation potential of human dental pulp stem cells (hDPSCs) toward Schwann cells, together with their functional capacity with regard to myelination and support of neurite outgrowth *in vitro*. Successful Schwann cell differentiation was confirmed at the morphological and ultrastructural level by transmission electron microscopy. Furthermore, compared to undifferentiated hDPSCs, immunocytochemistry and ELISA tests revealed increased glial marker expression and neurotrophic factor secretion of differentiated hDPSCs (d-hDPSCs), which promoted survival and neurite outgrowth in 2-dimensional dorsal root ganglia cultures. In addition, neurites were myelinated by d-hDPSCs in a 3-dimensional collagen type I hydrogel neural tissue construct. This engineered construct contained aligned columns of d-hDPSCs that supported and guided neurite outgrowth. Taken together, these findings provide the first evidence that hDPSCs are able to undergo Schwann cell differentiation and support neural outgrowth *in vitro*, proposing them to be good candidates for cell-based therapies as treatment for peripheral nerve injury.—Martens, W., Sanen, K., Georgiou, M., Struys, T., Bronckaers, A., Ameloot, M., Phillips, J., Lambrichts, I. Human dental pulp stem cells can differentiate into Schwann cells and promote and guide neurite outgrowth in an aligned tissue-engineered collagen construct *in vitro*.

A variety of traumas and diseases can cause peripheral nerve injury (PNI), which often results in chronic pain and disability (1). Endogenous repair is known to initiate after injury and is strongly dependent on the contribution of Schwann cells, as the regenerative capacity of peripheral nerves is reduced in their absence (2). Schwann cells not only reconstitute myelin, which is essential for fast neural action potential propagation, but also provide physical guidance (bands of Büngner) and trophic support for axonal regeneration. Regeneration following nerve transection is limited by the distance between the nerve stumps, and bridging strategies are required where direct end-to-end repair is not feasible. Bridging strategies include the use of tubes and decellularized nerve tissue, and, for longer gaps (>3 mm), the nerve autograft is currently regarded as the gold standard (1). While autografts provide Schwann cells and appropriate architecture for regeneration, there are problems with availability and donor site morbidity, and overall clinical outcomes show limited success (1, 3).

A wide range of biomaterial and tissue engineering approaches have been used to generate potential alternatives that recreate beneficial aligned cellular features of the autograft (4–6). In particular, hydrogels made from natural proteins have gained significant interest due to their functional extracellular matrix properties, inherent biocompatibility, and suitability as carriers for different cell types (7, 8); however, there are limitations associated with the generation and maintenance of guidance architecture in hydrogels (9). A technique was recently developed to align and stabilize Schwann cells and collagen fibrils in a collagen type I hydrogel, thereby generating an aligned tissue-like cellular biomaterial for neural tissue engineering (10). For this approach to be clinically useful, a suitable source of Schwann cells is required for the engineered neural tissue (EngNT) construct. The use of autologous Schwann cells for PNI is restricted because their isolation requires resection of another peripheral nerve, and they are known to expand slowly when cultured *in vitro*, thereby leading to the need for alternative cell sources (11, 12).

Adult stem cells, such as mesenchymal stem cells (MSCs), are promising candidates to treat PNI. MSCs can be isolated from a wide range of tissues and have been shown to secrete neurotrophic factors (NFs) capable of inducing axonal outgrowth, and they can differentiate into Schwann-like cells or neurons (13, 14). A promising alternative cell source is human dental pulp stem cells (hDPSCs; ref. 15). These are ectoderm-derived stem cells, originating from migrating neural crest cells and possessing MSC properties (16–19). The resulting stem cell population can be easily isolated from discarded wisdom teeth without the need for invasive tissue harvest associated with other sources of MSCs. Furthermore, their stem cell properties are retained after cryopreservation, providing the opportunity to establish a stem cell bank (15, 20). In addition to their ability to differentiate into cells of mesodermal lineages, hDPSCs have the potential to differentiate along the neural lineage. Even in an undifferentiated state, hDPSCs already express neural markers like S100, β-III-tubulin, and nerve growth factor receptor p75 and are able to produce and secrete a range of NFs, ciliary neurotrophic factor (CNTF), vascular endothelial growth factor (VEGF), brain-derived neurotrophic factor (BDNF), glia-derived neurotrophic factor (GDNF), and nerve growth factor b (b-NGF), thereby enhancing and guiding axonal outgrowth (21–25). Although several groups have already reported *in vitro* neuronal differentiation of hDPSCs (26–28), the differentiation of hDPSCs toward Schwann cells has not been reported to date.

Here we established a protocol for glial differentiation of hDPSCs *in vitro* and assessed the functional capacity of differentiated hDPSCs (d-hDPSCs) with regard to myelination and support of neurite growth. Our findings provide the first evidence that hDPSCs are able to undergo differentiation toward Schwann-like cells that support neural outgrowth *in vitro*, revealing them to be good candidates for cell-based therapies to treat PNI.

## MATERIALS AND METHODS

### Materials and products

All products were purchased from Sigma-Aldrich (Bornem, Belgium) unless stated otherwise.

### Isolation and differentiation of hDPSCs into Schwann-like cells

Human third molars were collected with written informed consent from donors (15–20 yr of age) undergoing tooth extraction for orthodontic or therapeutic reasons at Ziekenhuis Oost-Limburg (Genk, Belgium). When donors were underaged, written informed consent was obtained *via* their legal guardians. This study was approved by the medical ethical committee of Hasselt University. hDPSCs were isolated from dental pulp tissue *via* the explant method previously described (29). hDPSCs were maintained in minimal essential medium, α modification (αMEM) supplemented with 10% fetal bovine serum (FBS, Biochrom AG, Berlin, Germany), 2 mM l-glutamine, 100 U/ml penicillin, and 100 μg/ml streptomycin. When 80–90% confluency was reached, cells were routinely subcultured.

At passage 2–3, Schwann cell differentiation was induced by changing the medium to standard culture medium without FBS containing 1 mM β-mercaptoethanol (BME) for 24 h. Subsequently cells were incubated in standard culture medium supplemented with 35 ng/ml all *trans*-retinoic acid (RA). After 72 h, medium was changed to standard culture medium supplemented with 5 μM forskolin, 10 ng/ml basic fibroblast growth factor (b-FGF), 5 ng/ml platelet-derived growth factor AA (PDGFaa), and 200 ng/ml heregulin-β-1 (NRG) (Immunotools, Friesoythe, Germany). The cells were cultured in this supplemented medium for 2 wk with medium changes every 2–3 d (30). hDPSCs differentiated toward Schwann-like cells are henceforth referred to as d-hDPSCs. Both hDPSCs and d-hDPSCs were grown at 37°C in a humidified atmosphere with 5% CO_2_.

### Collection of conditioned medium

hDPSCs were seeded at a density of 20,000 cells/cm^2^ in standard hDPSC culture medium. After 24 h, hDPSCs were rinsed with PBS and incubated with standard culture medium supplemented with 0.1% FBS instead of 10% FBS. After 48 h of incubation, the medium was collected and stored at −80°C. Furthermore, conditioned medium from d- hDPSCs was collected as described above and stored at −80°C.

### Isolation of primary Schwann cells

Primary Schwann cells were isolated from postnatal d 17 Sprague-Dawley rats as described previously (31) with minor modifications. Briefly, sciatic nerves were dissected out and stripped free of epineurium. Following enzymatic treatment with 0.25% collagenase and 0.25% trypsin-EDTA, the nerve segments were mechanically dissociated. Cells from 2 nerve segments (1 animal) were seeded in a T25 flask precoated with 10 μg/ml poly-l-lysine. Further purification of the culture was performed as previously described (31).

### Immunocytochemistry

An immunocytochemical analysis was performed on hDPSCs, d-hDPSCs and primary Schwann cells with antibodies against glial fibrillary acidic protein (GFAP; 1:400; Leica Microsystems/NovoCastra, Diegem, Belgium), p75 (1:50, Dakocytomation, Glostrup, Denmark), laminin (1:1000, Abcam, Cambridge, UK), CD104 (1:100, Abcam), and nestin (1:500, Millipore, MA, USA) to determine the immunophenotype after differentiation. Cells were fixed with 4% paraformaldehyde (PFA) at 4°C for 20 min and washed with PBS. In case of intracellular targets, cells were permeabilized with Triton-X 0.05% for 30 min at 4°C. To block nonspecific binding sites, cells were incubated with 10% normal donkey serum at room temperature for 20 min. After washing with PBS, cells were incubated with primary antibody for 1 h, followed by incubation with donkey anti-rabbit Alexa488 (1:500) or donkey anti-mouse Alexa555 (1:500) secondary antibodies for 30 min at room temperature. Slides were mounted using 4′,6-diamidino-2-phenylindole (DAPI) with Prolong Gold Antifade (Molecular Probes, Merelbeke, Belgium). Fluorescence was visualized with a Nikon Eclipse 80i fluorescence microscope equipped with a Nikon DS-2MBWc digital camera (Nikon, Tokyo, Japan). Samples in which primary antibodies were omitted were used as a negative control.

### Ultrastructural analysis: transmission electron microscopy (TEM)

Following fixation with 2% glutaraldehyde (Laborimpex, Brussels, Belgium) in 0.05M cacodylate buffer (pH 7.3; Aurion, Wageningen, the Netherlands) at 4°C, the fixative was gently aspirated with a glass pipette, and the cells were postfixed in 2% osmium tetroxide (Aurion) for 1 h. Subsequently, the cell-seeded coverslips were put through a dehydrating series of graded concentrations of acetone and embedded in araldite according to the popoff method (32). Ultrathin sections (0.06 μm) were mounted on 0.7% formvar-coated copper grids (Aurion), contrasted with 0.5% uranyl acetate and a stabilized solution of lead citrate (both from Laurylab, Saint-Fons Cedex, France), and examined in a Philips EM 208 transmission electron microscope (Philips, Eindhoven, The Eindhoven) operated at 80 kV. The microscope was provided with a Morada Soft Imaging System (SIS; Olympus, Tokyo, Japan) camera to acquire high-resolution images of the evaluated samples. The images were processed digitally with iTEM-FEI software (Olympus SIS).

### Enzyme-linked immunosorbent assay (ELISA)

ELISAs were performed on conditioned medium derived from hDPSCs and d-hDPSCs in order to determine the concentration of BDNF, GDNF, neurotrophin 3 (NT-3) and b-NGF (RayBiotech, Boechout, Belgium). Experiments were performed in triplicate and absorbance was measured at 450 nm by means of the FLUOstar Optima multifunctional microplate reader (BMG Labtech, Ortenberg, Germany). Conditioned medium from 9 different donors was used, and ELISA tests were performed according to the manufacturer's protocol.

### Neonatal dorsal root ganglion (DRG) neuron cell cultures: 2-dimensional (2D) experiments

Experimental procedures involving neonatal animals were approved by the Hasselt University animal ethics advisory group. DRGs were harvested from 5-d-old Sprague-Dawley rat pups. Briefly, isolated DRG explants were dissociated with 0.025% collagenase at 37°C for 1 h. Dissociated cells were seeded onto coverslips at a density of 25,000 cells/cm^2^. Coverslips were precoated with poly-l-lysine (10 μg/ml) for 1 h. At 1–2 h after cell plating, medium was changed to remove nonadhering cells. Neurons were cultured for 24 h in DMEM/F12 medium supplemented with Glutamax, 10% FBS, 100 U/ml penicillin, and 100 μg/ml streptomycin.

### Survival assay

Isolated neonatal DRG cultures were seeded in a 96-well plate at a density of 10,000 cells/well. The next day, cells were washed twice with PBS, and the medium was replaced with conditioned medium from hDPSCs or d-hDPSCs. αMEM with 10 or 0.1% FBS (henceforth DRG 0.1%) was used as positive or negative control, respectively. After 48 h, the medium was removed, and 500 μg/ml MTT was added to the wells. The MTT solution was removed after an incubation time of 4 h at 37°C. A mixture of 0.01 M glycine and DMSO was added to each well. The absorbance was measured at a wavelength of 540 nm with a Benchmark microplate reader (Bio-Rad Laboratories, Nazareth Eke, Belgium). Conditioned medium from cultures from ≥7 different donors was used.

### Neurite regeneration assay

Neonatal DRG cultures were incubated with conditioned medium from hDPSCs or d-hDPSCs to analyze the effect on neurite outgrowth. αMEM with 10% FBS or DRG 0.1% was used as positive or negative control, respectively. After 48 h, cells were fixed in 4% PFA and immunostained using anti-β-III-tubulin. Four independent experiments were carried out, and neurite outgrowth was assessed by measuring the length of the longest neurite of individual cells.

### Adult DRG cell cultures: 3-dimensional (3D) experiments

Experimental procedures involving adult animals were conducted in accordance with the UK Animals (Scientific Procedures) Act (1986) and approved by the Open University animal ethics advisory group. Dissociated DRG cultures were prepared from adult (200–300 g) Sprague-Dawley rats that were culled using CO_2_ asphyxiation. Briefly, spines were removed, and DRG explants were collected in DMEM with 10% FBS and 100 U/ml penicillin and 100 μg/ml streptomycin. After removal of the connective tissue and nerve roots, DRGs were placed into a collagenase solution (0.125%) at 37°C. After 90 min of incubation, collagenase was removed, and explants were triturated in DMEM with 10% FBS until a homogenous cell suspension was obtained. Next, cells were transferred to culture flasks containing DMEM with 10% FBS, 100 U/ml penicillin, 100 μg/ml streptomycin, and 10 μM cytosine arabinoside (Ara-C) for 24 h to deplete the non-neuronal cells.

### Myelination in hydrogel

d-hDPSC-seeded collagen gels were tethered within rectangular stainless steel molds according to methods described previously (33–35). Gels were prepared using 10% cell suspension (a mixture of 250,000 d-hDPSCs and 5 dissociated DRG explants per milliliter of gel) mixed with 10% MEM and 80% type I rat tail collagen (5 mg/ml in 0.6% acetic acid; First Link, Wolverhampton, UK) following neutralization using sodium hydroxide. This mixture (1 ml) was added to each mold at 4°C and integrated with tethering mesh at opposite ends before setting at 37°C for 10 min. Tethered gels were immersed in medium, and after 2 wk, the contracted hydrogels were fixed overnight with 4% PFA at 4°C. The higher collagen concentration (compared to tethered gels used in EngNT assembly) delayed the process of hydrogel contraction and cell alignment, thereby allowing long-term incubation of the uncompressed cellular hydrogel in the mold.

### TEM of cellular hydrogels

Cellular hydrogels were fixed with 4% PFA and processed for TEM as described above, with the ultrathin sections sliced perpendicular to the longitudinal axis of the hydrogel in this case. The total numbers of d-hDPSCs and neurites were manually counted in each ultrathin section (3 sections/gel, *n*=5 gels). A distance of <40 nm between a neurite and a d-hDPSC was considered to be a contact between cell types. Data were normalized to number per square millimeter of hydrogel section.

### Generation of EngNT using d-hDPSCs

A cell-seeded collagen gel was prepared as described above, with some modifications. d-hDPSCs were suspended in a collagen type I solution of 2 mg/ml (First Link) to give a final density of 10^6^ cells/ml gel mixture. Tethered gels were immersed in medium for 4–6 h to allow alignment to develop. Aligned cellular gels were stabilized by plastic compression: Aligned tethered gels were separated from the tethering mesh using a scalpel, then immediately compressed by loading the gel with 120 *g* for 1 min, while fluid was removed into a porous paper pad underneath. The resulting sheets of EngNT were transferred to 24-well plates for *in vitro* neurite growth experiments.

### Seeding of dissociated DRG neurons on top of EngNT

Adult DRG neurons (20 dissociated DRG explants) were seeded onto the surface of each EngNT sheet and allowed to settle for 30 min, and then constructs were immersed in medium at 37°C in a humidified incubator with 5% CO_2_. After 3 d, the EngNT-neuron cocultures were washed briefly in PBS and fixed in 4% PFA at 4°C for 24 h, followed by immunofluorescence staining as described previously for collagen gels (36, 37), to detect β-III-tubulin positive neurons and S100-positive Schwann cells.

### Analysis of angle of deviation

Confocal microscopy (Leica SP5) was used in the assessment of d-hDPSC alignment in EngNT, and d-hDPSC and neurite alignment and growth in the EngNT-neuron cocultures. Four equivalent fields were analyzed per gel using a predetermined sampling protocol. The total area sampled per gel was 0.49 mm^2^. Images were captured using a ×40 oil immersion lens, and *z*-stacks were 20 μm, with a step size of 1 μm. Image analysis was conducted using Volocity software (PerkinElmer, Waltham, MA, USA) running automated 3D image analysis protocols to measure the angle of Schwann cell alignment and neurite alignment in each field. To evaluate the directional growth of neurites on EngNT containing d-hDPSCs, angles of neurites deviating from the mean d-hDPSC angle in each field were measured.

### Statistical analysis

Statistical analysis was performed using GraphPad Prism 5 software (GraphPad, San Diego, CA, USA). Data from the survival assay and neurite outgrowth were first controlled for normality by means of a D'Agostino-Pearson omnibus normality test, followed by comparison of control and experimental groups by means of a Kruskal-Wallis test while applying a Dunn's multiple comparison *post hoc* test. Data from ELISA tests were submitted to a D'Agostino-Pearson omnibus normality test, followed by an unpaired *t* test. Data from the proliferation assays were compared by means of a 2-way ANOVA followed by Bonferroni's multiple comparison test. Values of *P* ≤ 0.05 were considered statistically significant. All data are expressed as means ± sem.

## RESULTS

### Morphology and immunophenotype of d-hDPSCs *in vitro*

**Figure 1.**
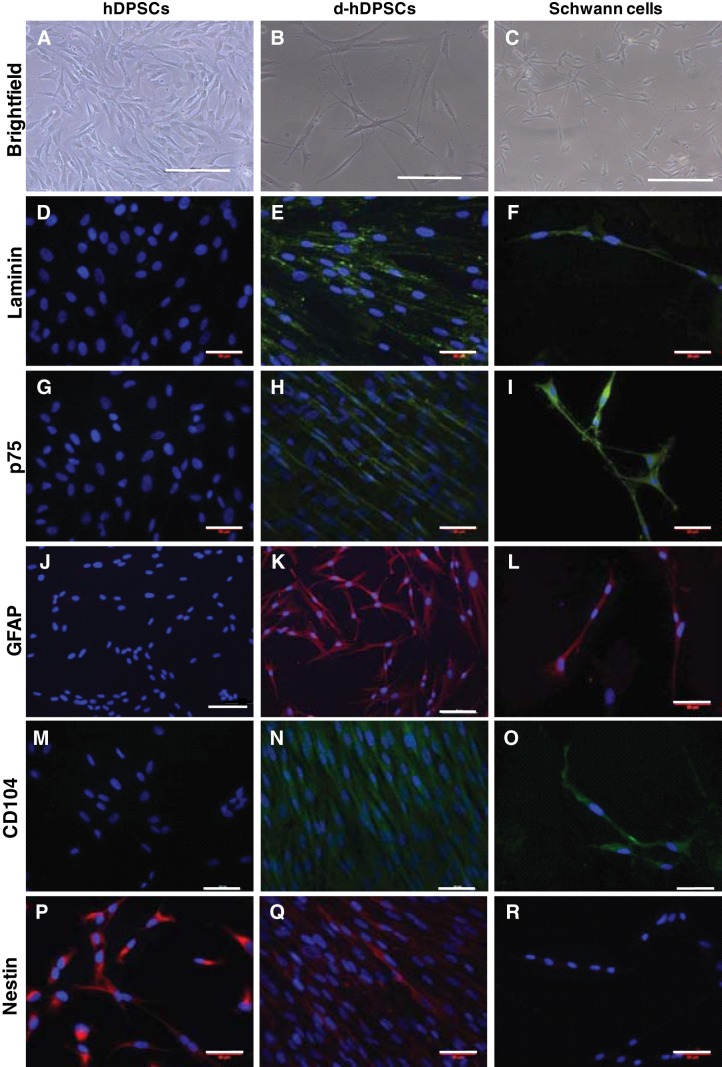
Phenotyping hDPSCs after Schwann cell differentiation. Brightfield imaging (*A–C*) and immunocytochemistry (*D–R*) were performed on hDPSCs (*A*, *D*, *G*, *J*, *M*, *P*) and d-hDPSCs (*B*, *E*, *H*, *K*, *N*, *Q*) for the typical Schwann cell markers laminin (*D–F*), p75 (*G–I*), GFAP (*J–L*), CD104 (*M–O*), and nestin (*P–R*). Nuclei were counterstained with DAPI (blue). Primary rat Schwann cells (*C*, *F*, *I*, *L*, *O*, *R*) were used as positive controls for differentiation. Scale bars = 200 μm (*A–C*); 50 μm (*D–I, L–R*); 100 μm (*J, K*).

Schwann cell differentiation was induced in hDPSCs at passage 2–3. *In vitro*, undifferentiated hDPSCs displayed a flattened fibroblast-like morphology (**Fig. 1*A***). After 24 h in differentiation medium containing 1 mM BME, cells adopted an elongated shape. Following the induction protocol, cells acquired a bipolar cell morphology resembling primary rat Schwann cells in culture (Fig. 1*B*, *C*).

To evaluate the expression of glial markers, immunocytochemical staining was performed with antibodies against laminin, p75, GFAP, CD104, and nestin in hDPSCs, d-hDPSCs, and Schwann cells. Both Schwann cells and d-hDPSCs showed a positive immune reaction for laminin, p75, GFAP, and CD104 (Fig. 1*D–O*). Furthermore, expression of the early neural marker nestin decreased in differentiated cell cultures compared to hDPSCs and was not detected in Schwann cells (Fig. 1*P–R*).

**Figure 2.**
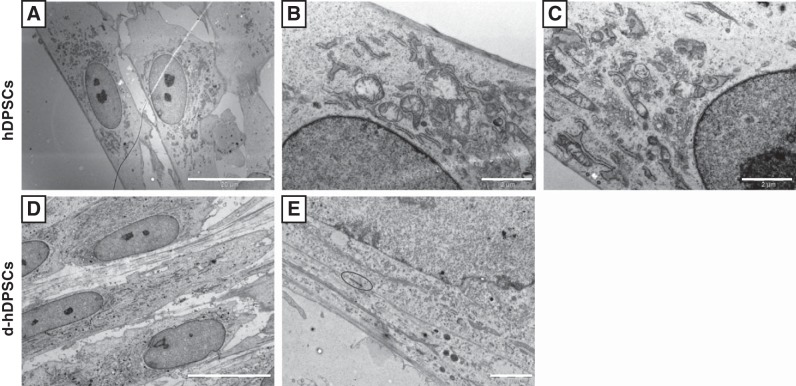
Transmission electron micrographs of hDPSCs (*A–C*) and d-hDPSCs (*D, E*). Ultrastructural characteristics of d-hDPSCs differ markedly from those of hDPSCs, with cell-cell contacts (*E,* oval) between neighboring cells. Scale bars = 20 μm (*A, B, D*); 2 μm (*C, E*).

At the ultrastructural level, hDPSCs and d-hDPSCs displayed large euchromatic nuclei with prominent nucleoli. Within the cytoplasm of hDPSCs, a perinuclear zone was observed containing organelles such as mitochondria, rough endoplasmatic reticulum cisternae, and some Golgi apparatus (**Fig. 2*A***–***C***). In cultures of d-hDPSCs, however, organelles were spread throughout the cytoplasm, and cell-cell contacts were often visible between neighboring cells (Fig. 2*D*, *E*; oval).

### Secretion of neurotrophic factors by hDPSCs and d-hDPSCs

**Figure 3.**
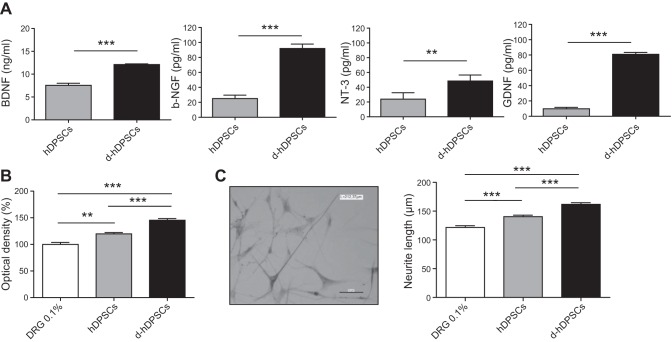
Neurotrophic factor secretion. A) ELISAs indicated a significant increase in BDNF, b-NGF, NT-3, and GDNF levels after differentiation (*n*=9). *B*, *C*) Neural survival (*B*; *n*=7) and neurite outgrowth (*C*; *n*=4) were significantly improved with conditioned medium from hDPSCs and d-hDPSCs, with the latter being more potent. Scale bar = 50 μm. Data represent means ± sem. **P < 0.01, ***P < 0.001.

The concentration of GDNF, BDNF, NT-3, and b-NGF, secreted in the conditioned medium of hDPSCs and d-hDPSCs, was determined by means of ELISA (**Fig. 3*A***). The levels of BDNF, b-NGF, NT-3, and GDNF were significantly increased after differentiation by 1.60-, 3.68-, 2.02-, and 8.27-fold, respectively.

DRG cultures were incubated with conditioned medium of hDPSCs or d-hDPSCs for 48 h. MTT assays showed a significant increase in the survival of DRG cells after the addition of conditioned medium from hDPSCs and d-hDPSCs (Fig. 3*B*). Furthermore, compared with hDPSCs, a higher percentage of survival was observed in DRG cultures after the addition of conditioned medium from d-hDPSCs.

To detect the outgrowth of neurites in DRG cultures following 48 h of incubation with conditioned medium of hDPSCs and d-hDPSCs, immunocytochemical staining for β-III-tubulin was performed. A significant increase in the length of the longest neurite was observed after adding conditioned medium of hDPSCs and d-hDPSCs (Fig. 3*C*). In addition, conditioned medium of d-hDPSCs induced a distinct positive influence on neurite outgrowth compared to conditioned medium of undifferentiated hDPSCs.

### d-hDPSCs are aligned in EngNT and guide neurite outgrowth *in vitro*

**Figure 4.**
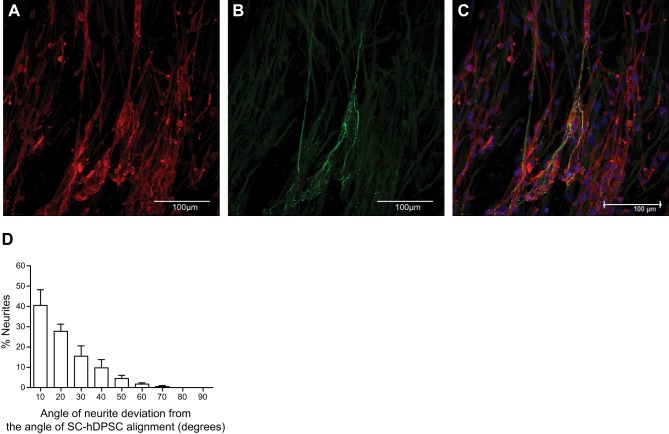
d-hDPSC alignment in EngNT guides neurite outgrowth in coculture. *A–C*) Confocal images represent d-hDPSCs (S100, red) and neurites (b-III-tubulin, green). Nuclei were counterstained with DAPI (blue). Scale bars = 100 μm. *D*) Frequency distribution of neurite angles compared to mean angle of d-hDPSC alignment in each field (field volume: 9.8×10^6^ μm^3^). Data represent means ± se (in 10° bins; *n*=4).

The capacity of d-hDPSCs to contribute toward a beneficial neuroregenerative environment was assessed *in vitro*. After d-hDPSCs aligned within tethered collagen gels within 4–6 h, constructs were stabilized by removal of interstitial fluid from the hydrogel. This process did not affect d-hDPSC survival (data not shown). Columns of aligned d-hDPSCs within the EngNT were visualized by means of immunofluorescence and confocal microscopy (**Fig. 4*A***). When dissociated DRG neurons were cultured on top of this aligned cellular material for 3 d, neurite extension along aligned d-hDPSCs was observed (Fig. 4*B–C*). The relative frequency distribution of neurite deviation from the mean d-hDPSCs orientation revealed that the majority of neurites (84±11%) deviated with an angle of only 0–30° from the underlying d-hDPSC columns (Fig. 4*D*). Neurites growing near perpendicular (80–90°) to the overall direction of aligned d-hDPSCs were not observed.

### Ultrastructural evaluation of myelination capacity of d-hDPSCs

**Figure 5.**
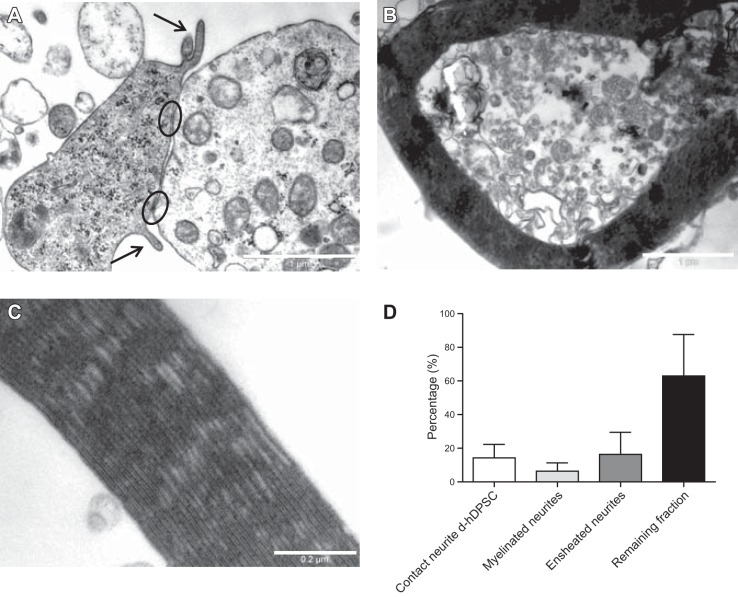
Ultrastructural analysis of myelination capacity of d-hDPSCs. *A*) Multiple cell-cell contacts (encircled) and enfoldment of neurites by pseudopodial processes of d-hDPSCs (arrows) were frequently observed. *B–C*) Myelin sheaths were present in the culture (*B*), showing typical periodic and intraperiodic lines (*C*). *D*) Total number of d-hDPSCs and neurites per square millimeter of hydrogel. Scale bars = 1 μm (*A, B*); 0.2 μm (*C*).

Three-dimensional cocultures of d-hDPSCs and DRG-derived primary neurons in EngNT were established to assess the myelination capacity of d-hDPSCs. Cytoplasm of both cell types could be easily distinguished based on differential amounts of cell organelles. Neurites were characterized by numerous mitochondria, whereas d-hDPSC cytoplasm was mainly occupied by rough endoplasmic reticulum and free ribosomes. Proximity of d-hDPSCs and neurons resulted in an extensive number of cell-cell contacts (**Fig. 5*A***). Neurites were engulfed by d-hDPSCs following enfoldment using pseudopodial processes. d-hDPSCs produced myelin sheaths ranging in thickness from 0.1 to 1 μm (Fig. 5*B*). Once established, myelin sheaths contained multiple neurites and exhibited typical periodic and intraperiodic lines (Fig. 5*C*). Manual counting of the total number of d-hDPSCs and neurites showed that, on average, 20 d-hDPSCs and 172 neurites/mm^2^ hydrogel were present. The total number of neurites consisted of 4 subgroups: neurites that were in contact with d-hDPSCs (14.26%), myelinated neurites (6.42%), neurites that were ensheathed by the cytoplasm of d-hDPSCs (16.40%), and the remaining fraction showing none of the previous characteristics (62.93%) (Fig. 5*D*).

## DISCUSSION

In the present study, we established a protocol for the differentiation of hDPSCs into cells with a Schwann cell phenotype and functionality *in vitro*. Although several researchers described the possible differentiation of bone marrow-derived MSCs or adipose-derived MSCs toward Schwann cells (11, 38, 39), this is the first time, to our knowledge, that successful differentiation of hDPSC toward a Schwann cell phenotype has been established and well characterized. Following the differentiation protocol, hDPSCs expressed typical Schwann cell markers and promoted neuronal survival and neurite outgrowth. Moreover, in a 3D coculture model with neurons, d-hDPSCs guided neurite outgrowth and were able to myelinate neurites extended by DRG neurons.

Since DPSCs originate from migrating neural crest cells, they are thought to be predisposed toward differentiating into peripheral glial cells under the correct environmental conditions (16–19).

In addition to this neural crest lineage, the high proliferation capacity, multipotency, plasticity, and immunomodulatory properties of DPSCs make them excellent candidates for regenerative medicine purposes, especially in the field of neural tissue engineering (22, 27, 40, 41).

Differentiation of hDPSCs toward Schwann-like cells was induced *in vitro* by the addition of various factors: BME, RA, and growth media supplemented with a cocktail of growth factors containing PDGFaa, b-FGF, forskolin, and NRG. BME is known to promote the formation of neurite-like outgrowth (42, 43) as is seen in cell cultures after 24 h administration of BME. RA was used to further induce morphological cell changes, as several reports state that RA together with BME can work as a triggering factor that alters cell morphology. Furthermore, RA induces differentiation of embryonic stem cells into neural cells and regulates the expression of transcription factors that play a role in neural cell determination (30, 44). An increase in cAMP, and thus an elevated expression of mitogenic genes, can be achieved when cells are treated with forskolin. Taken together, BME and RA have altered cell morphology, and further use of forskolin, b-FGF, PDGFaa, and NRG synergistically promote the differentiation of hDPSC into cells with Schwann cell characteristics.

The ultrastructural characteristics of undifferentiated hDPSCs have previously been described (29). As seen in our cultures, undifferentiated hDPSCs are fibroblast-like cells containing a perinuclear organelle-rich zone and a peripheral zone lacking any cell organelles. Furthermore no cell-cell contacts and extracellular matrix components were observed (29). After differentiation, we observed that d-hDPSCs adopted a spindle-shaped bipolar morphology with numerous organelles spread throughout the cell cytoplasm.

To confirm the Schwann cell-like phenotype of differentiated hDPSCs, the expression of multiple markers was evaluated. In order for MSCs to develop toward a neural cell lineage, nestin expression is essential (45). On differentiation, nestin expression is known to decrease, which is in accordance with the lower levels of nestin seen in d-hDPSCs compared to undifferentiated hDPSCs. Whereas laminin, p75, GFAP, and CD104 expression was hardly detectable in undifferentiated hDPSCs, d-hDPSCs stained strongly positive for these glial markers, similar to Schwann cells. CD104, also known as integrinβ4, associates with integrinβ6 to form an adhesion receptor for laminins and is widely expressed by Schwann cells. These results demonstrate the successful morphological differentiation of hDPSCs toward Schwann-like cells.

Previous studies have demonstrated that DPSCs secrete an array of NFs both *in vitro* and *in vivo* and that different neuronal populations exhibit enhanced neuronal survival and neurite outgrowth in the presence of these DPSCs-derived NFs (23–25, 46). In line with these findings, we observed the secretion of GDNF, BDNF, NT-3, and b-NFG by undifferentiated hDPSCs and their beneficial effects on neuronal survival and neurite length of cultured dissociated DRG neurons. As denervated Schwann cells are known to secrete NFs, and axonal regeneration is reduced in the absence of Schwann cells (47), we predicted that d-hDPSCs would have better neuroprotective and neurotrophic effects than hDPSC. Indeed, not only did significantly more DRG neurons survive with the use of conditioned medium derived from d-hDPSC cultures compared to undifferentiated hDPSCs cultures; an increase in neurite length was also observed.

In the field of neural regeneration, different conduits (natural, synthetic, resorbable, and nonresorbable hydrogels or polymers), in all cases being hollow tubes, have been evaluated to provide guidance for regrowing axons (1). Although promising results are obtained, these conduits lack the microarchitecture typical of nerve tissue (48). For optimal nerve regeneration across a long gap, it is important to use a scaffold that mimics key features of the native environment: Schwann-like cells secreting neurotrophic factors (like denervated Schwann cells in an autograft), Schwann-like cells organized into aligned columns (like bands of Büngner), and Schwann-like cells surrounded by an aligned nanofibrillar collagen extracellular matrix (like the endoneurium).

Since our results showed that hDPSC were able to differentiate into Schwann-like cells and were capable of secreting NFs that play a role in neural regeneration, we evaluated whether d-hDPSCs were able to self-align in 3D collagen to form EngNT, and whether they could provide guidance for regrowing axons.

After seeding d-hDPSCs in a collagen type I hydrogel, alignment of d-hDPSCs was obtained, and this was stabilized using plastic compression to form EngNT, as previously shown using Schwann cells (8). This organization of aligned d-hDPSCs in a collagen matrix resembled the bands of Büngner, which are important for accurate guiding of axonal regeneration. Indeed, it has previously been shown that in the process of nerve regeneration, the organization of Schwann cells into columns (bands of Büngner) is of critical importance to guide and support axonal regeneration from the proximal to the distal end of the injury (48). Cells in a collagen gel will form stable integrin bonds with the collagen fibrils, and then cytoskeletal activity generates forces that contract the gel. EngNT formation as used here exploits this phenomenon; anchoring the ends of a rectangular cellular collagen gel results in development of a longitudinal axis of tension, causing cell elongation and alignment of cells and collagen fibers (8–10).

The majority of DRG neurites seeded on top of EngNT acquired the direction of aligned d-hDPSCs. In this manner, d-hDPSCs provided a strong guidance cue for neurite growth, which is necessary to promote neural regeneration *in vivo*. Our results were similar to previous studies where aligned glial cell environments supported and guided neurite growth *in vitro* (10, 49–51). The aligned cells rather than the collagen matrix are likely to be the main contributor to the promotion of neurite growth, since a previous study showed that little outgrowth was detected in decellularized and acellular constructs compared to EngNT containing live Schwann cells (10).

Tethered hydrogels were used to establish 3D cocultures of DRG neurons and d-hDPSCs to investigate the myelination capacity of the latter. Although the majority of neurites were not myelinated, a close apposition between cellular extensions of d-hDPSCs and neurons was frequently observed. Often these areas were characterized by the presence of multiple cell junctions, which, in turn, might represent the initiating step of the myelination process (52). The *in vitro* model used here showed that when neurons and d-hDPSCs were cultured together within a 3D hydrogel environment, the d-hDPSCs spontaneously ensheathed neurites and, in some cases, generated myelin structures, both characteristics that would be associated with neuron-Schwann cell interactions *in vivo*.

In the present study, hDPSCs were successfully differentiated toward Schwann-like cells both on the morphological and functional level. d-hDPSCs expressed characteristic Schwann cell markers, promoted neurite outgrowth in 2D and 3D culture environments, and exhibited typical Schwann cell interactions with neurons, such as ensheathment and myelination of neurites. These features, coupled with their potential clinical availability and utility as a component of tissue engineered constructs, make hDPSCs promising candidates for investigation as cell-based therapies in peripheral nerve injury.
